# Rationalization of paclitaxel insensitivity of yeast β-tubulin and human βIII-tubulin isotype using principal component analysis

**DOI:** 10.1186/1756-0500-5-395

**Published:** 2012-08-01

**Authors:** Lalita Das, Bhabatarak Bhattacharya, Gautam Basu

**Affiliations:** 1 Department of Biochemistry, Bose Institute, P-1/12 CIT Scheme VIIM, Kolkata, 70054, India; 2 Department of Biophysics, Bose Institute, P-1/12 CIT Scheme VIIM, Kolkata, 70054, India

## Abstract

**Background:**

The chemotherapeutic agent paclitaxel arrests cell division by binding to the hetero-dimeric protein tubulin. Subtle differences in tubulin sequences, across eukaryotes and among β-tubulin isotypes, can have profound impact on paclitaxel-tubulin binding. To capture the experimentally observed paclitaxel-resistance of human βIII tubulin isotype and yeast β-tubulin, within a common theoretical framework, we have performed structural principal component analyses of β-tubulin sequences across eukaryotes.

**Results:**

The paclitaxel-resistance of human βIII tubulin isotype and yeast β-tubulin uniquely mapped on to the lowest two principal components, defining the paclitaxel-binding site residues of β-tubulin. The molecular mechanisms behind paclitaxel-resistance, mediated through key residues, were identified from structural consequences of characteristic mutations that confer paclitaxel-resistance. Specifically, Ala277 in βIII isotype was shown to be crucial for paclitaxel-resistance.

**Conclusions:**

The present analysis captures the origin of two apparently unrelated events, paclitaxel-insensitivity of yeast tubulin and human βIII tubulin isotype, through two common collective sequence vectors.

## Background

Microtubules are structures composed of polymerized tubulin heterodimers and play fundamental roles in vital cellular processes such as chromosome segregation, intracellular transport and maintenance of cell shape [1]. The major component of microtubules is the hetero-dimeric (αβ) protein tubulin. Mammalian tissues express different α- and β-tubulin isotypes [2] that are known to play specific biological functions, exhibit tissue-restricted expression, possess differential binding affinities for antimitotic agents and exhibit different kinetics and dynamics of microtubule assembly [3-5].

Paclitaxel, a product of plant secondary metabolism, binds to the β-tubulin subunit and inhibits microtubule dynamics, thereby blocking cell cycle progression during mitosis at the metaphase/anaphase transition and activating cell death [6-8]. It is an important cancer chemotherapeutic agent for treatment of advanced ovarian, lung and breast carcinoma, and shows promising activity against several other carcinomas [9,10]. Although paclitaxel is currently used in chemotherapy, clinical resistance against paclitaxel can become a significant problem. The human βIII tubulin isotype has been implicated to play a crucial role in conferring paclitaxel resistance. Increased expression of the βIII tubulin isotype inhibits cell proliferation and confers resistance to paclitaxel [11-13]. Increased relative abundance of the βIII isotype is also known to destabilize the microtubule [14] and augment paclitaxel resistance [13,15-17].

Similar to the βIII isotype of mammalian brain tubulin, tubulin from the budding yeast, *Saccharomyces cerevisiae*, shows weak binding affinity for paclitaxel [18,19] despite the fact that yeast tubulin shares 75% amino acid identity with mammalian brain tubulin. When five amino acid residues in yeast β-tubulin (A19K, T23V, G26D, N229H, and Y272F) were changed to the respective residues found in the mammalian brain tubulin, a paclitaxel-binding site could be created in yeast tubulin [20]. Of these, the effect from two (A19K and N229H) was shown to be negligible from single mutational studies [21]. The mutations changed only a handful of residues, yet there was a dramatic change in the paclitaxel binding affinity. This is analogous to the difference between βIII tubulin and other β-tubulin isotypes (they differ at only a few sequence positions) where a dramatic change in paclitaxel affinity is also observed.

Although the final effect of replacing βIII tubulin by other isotypes, and, mutating yeast tubulin at three specific positions — regeneration of paclitaxel-binding affinity — is the same, the underlying mechanisms may be different. Here we focus on deciphering such mechanisms, specifically identifying residues and their interactions, responsible for paclitaxel resistance of yeast tubulin and the βIII tubulin. To identify the role of the key residues, we analyzed β-tubulin sequences across a large number of eukaryotic families (animals, fungi, protists and plants) and performed principal component analysis (PCA) in the sequence-space defining the paclitaxel-binding site, obtained from the structure of paclitaxel-bound αβ-tubulin [22,23]. Projection of binding site residues on a plane defined by the two principal component (PC) axes identified two orthogonal ways by which residues varied across eukaryotes. While the paclitaxel resistance of yeast tubulin included contribution from both the axes, the paclitaxel resistance of the βIII isotype correlated with contribution from only one of the two axes. Analysis of structural consequences of modifying key residues allowed us to identify mechanisms of paclitaxel resistance of yeast and the βIII isotype. Principal component analysis also allowed us to predict the paclitaxel sensitivity of eukaryotic tubulin for which experimental data are unavailable.

## Results

### Multiple sequence alignment of primary paclitaxel-binding residues

**Figure 1:**
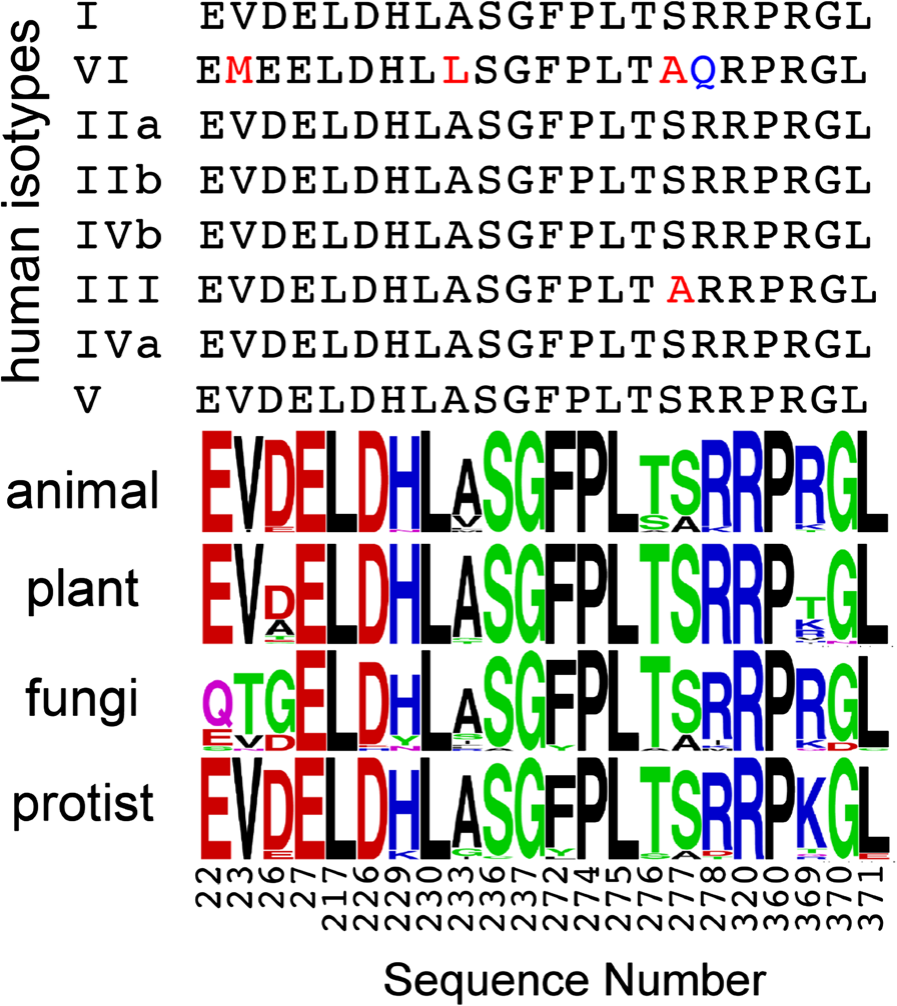
**Sequence variation at the paclitaxel-binding site (PBS) of human tubulin isotypes and β-tubulin sequences across eukaryotic families (shown as sequence logo plots).** Sequence numbering is consistent with that used in the pdb file 1JFF.

Instead of analyzing the entire tubulin sequence, we analyzed a sequence subset that is most likely to control paclitaxel binding. A total of 22 tubulin residues, occurring within 5 Å from paclitaxel in the paclitaxel-bound tubulin structure (pdb code: 1JFF) [23], were identified and defined as the paclitaxel binding site (PBS) residues. From a multiple sequence alignment using the program ClustalW [24], amino acids corresponding to the 22 PBS residues were identified in 125 eukaryotic β-tubulin sequences, as shown in Figure 1 (through out this work we have used sequence numbering used in the PDB entry 1JFF; this sequence numbering is slightly different from the original sequence numbering to accommodate sequence alignment of α and β–chains). To schematically depict sequence variations at these residue positions across the eukaryotic families, sequence logo [25] plots were constructed for each eukaryotic family, as depicted in Figure 1. Among the 22 residues, eight residues (27, 217, 230, 237, 274, 275, 320, and 360) were strictly conserved across eukaryotes, while the other 14 showed mild to strong variation. Among the 14 variable positions, two positions (23 and 26) correlated with mutational data that resulted in the non-binding of paclitaxel to yeast tubulin or microtubules [20]. The human β-tubulin isotypes were also multiple sequence aligned and the corresponding PBS residues were identified, as shown in Figure 1. Isotype III has one change (Ser277Ala) while isotype VI is characterized by a number of sequence variations (Val23Met, Ala233Leu, Ser277Ala and Arg278Gln). 

### Principal component analysis in the primary paclitaxel-binding sequence space

**Figure 2:**
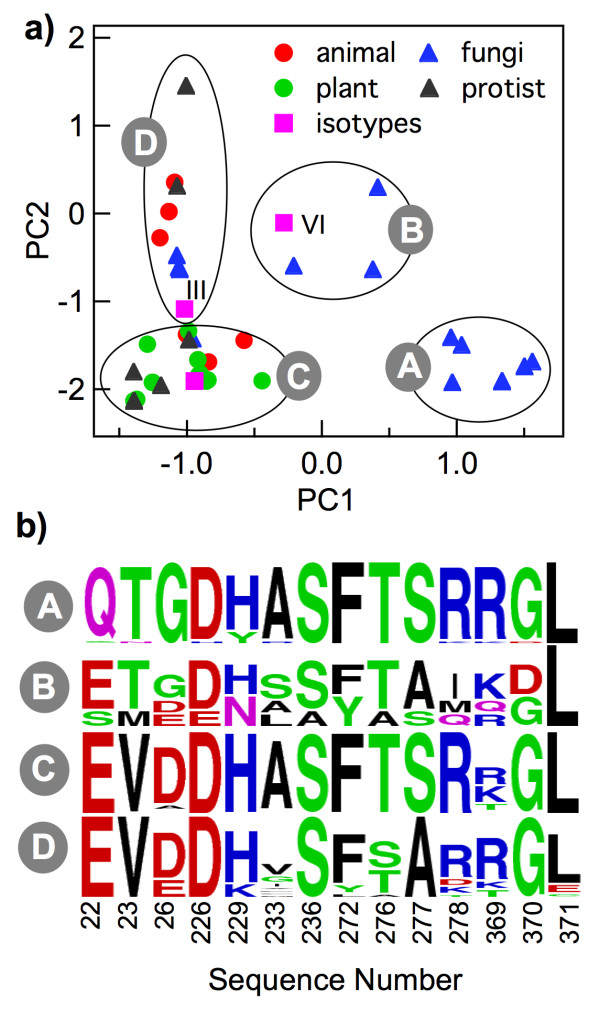
**Principal component analysis of β-tubulin PBS residues. a)** Projection of β-tubulin PBS on to a plane defined by the first and second principal component vectors. **b)** Sequence variations in each group (A, B, C and D) of panel **(a)** depicted as sequence logo plots.

The sequence variations in the PBS (Figure 1) can be further resolved by principal component analysis. In summary, PCA seeks vectors that reflect maximum change in residue variations, in a collective manner. Two such vectors, PC1 and PC2, associated with the highest mean square variations, were identified by PCA. The eukaryotic PBS sequences were then projected onto a plane defined by PC1 and PC2 (Figure 2a). Before analyzing Figure 2a, let us recapitulate what is known about experimental affinities of paclitaxel towards tubulin from different eukaryotic families (each eukaryotic family is marked by a different color in Figure 2a). Paclitaxel is known to interact with animal tubulin/microtubule [26]. Paclitaxel also interacts with plant microtubules — rose [27], maize and tobacco [28,29] are some examples. Paclitaxel is known to block the growth of *Leishmania donovani*, a protist, at low concentrations (<1 μM) [30]. However paclitaxel is insensitive to tubulin from fungi, the prime example being budding yeast *S. cerevisiae*[19]. However, the apparent eukaryotic family-specific paclitaxel-binding trait of β-tubulin is not reflected in Figure 2a. An examination of Figure 2a shows that there is no clear eukaryotic-family specific clustering, except that the PC1 projections of most fungal β-tubulins are positive. 

To understand Figure 2a better, we divided the projected PBS site residues into four groups (clusters) — A, B, C and D (see Tables 1, 2, 3, 4). Sequence alignments of members belonging to each group yielded four sequence logo plots, as shown in Figure 2b. These sequence logo plots represent the overall features of the respective groups. Dominant sequence changes, in going from one group to the other, either vertically or horizontally, are: T23V and G26D (horizontal move) and A277S (vertical move). Groups A and B exclusively contain members of the fungi family. Group C contains all members of the plant family and majority of the animal and the protist family. However, it also contains a fungal pathogen of maize, *Cochliobolus heterostrophus*. Group D contains members from all families except plants. Projection of human β-tubulin isotypes on the PC1-PC2 plane showed that all isotypes appear in group C except β-III (group D) and β-VI (group B).

**Table 1:** Animal β-tubulin sequences used in this work

**GI/NCBI Ref.**	**Organism**	**PBS residues**	**Group**
			**(Figure**2**)**
NM_178014.2	Human (isotype I)	EVDDHASFTSRRGL	C
NM_001069.2	Human (isotype IIa)	EVDDHASFTSRRGL	C
NM_178012.4	Human (isotype IIb)	EVDDHASFTSRRGL	C
NM_006088.5	Human (isotype IVb)	EVDDHASFTSRRGL	C
NM_006086.3	Human (isotype III)	EVDDHASFTARRGL	D
NM_006087.2	Human (isotype IVa)	EVDDHASFTSRRGL	C
NM_032525.1	Human (isotype V)	EVDDHASFTSRRGL	C
NM_030773.3	Human (isotype VI)	EMEDHLSFTAQRGL	B
66734014	*Ancylostoma duodenale*	EVDDHVSFSAKRGL	D
159161	*Haemonchus contortus*	EVDDHVSFSAKRGL	D
62836546	*Strongyloides stercoralis*	EVEDHMSFAARKGL	D
3046907	*Onchocerca volvulus*	EVDDHVSFSARRGL	D
3046903	*Dirofilaria immitis*	EVDDHVSFSARRGL	D
156096	*Brugia pahangi*	EVDDHVSFSARRGL	D
19773428	*Bombyx mori*	EIDDHLSFTSRRGL	C
16974673	*Saimiri sciureus*	EVDDHASFSSRRGL	C
159727	*Enteroctopus dofleini*	EVEDHASFTSRTGL	C
289741319	*Glossina morsitans*	EVDDHASFTSRKGL	C
135490	*Sus scrofa*	EVDDHASFTSRRGL	C
50844501	*Bos Taurus*	EVDDHASFTSRRGL	C
10242164	*Notothenia coriiceps*	EVDDHASFTSRRGL	C
24645350	*Drosophila melanogaster*	EVDDHASFTSRRGL	C
1335661	*Patella vulgata*	EVDDHASFTSRRGL	C
56603670	*Crassostrea gigas*	EVDDHASFTSRRGL	C
51860821	*Loligo pealei*	EVDDHASFTSRRGL	C
74136187	*Macaca mulatta*	EVDDHASFTSRRGL	C
90960962	*Pan troglodytes*	EVDDHASFTSRRGL	C
49481	*Cricetulus griseus*	EVDDNASFTSRRGL	C
6892	*Caenorhabditis elegans*	EVDDHASFTSRRGL	C
10242186	*Chionodraco rastrospinosus*	EVDDHASFTSRRGL	C
4558495	*Trichuris trichiura*	EVDDHASFTSRRGL	C
16974663	*Papio hamadryas*	EVDDHASFTSRRGL	C
1769528	*Heliothis virescens*	EVDDHASFTSRRGL	C
30088884	*Aplysia californica*	EVDDHASFTSRRGL	C
17402390	*Fasciola hepatica*	EVDDHASFTSRRGL	C
135489	*Paracentrotus lividus*	EVDDHASFTSRRGL	C
7838279	*Meriones unguiculatus*	EVDDHASFTSRRGL	C
7838199	*Echinococcus multilocularis*	EVDDHASFTSRRGL	C

Sequence numbers: 22-23, 26, 226, 229, 233, 236, 272, 276-278, 369-371.

**Table 2:** Fungi β-tubulin sequences used in this work

**GI/NCBI Ref.**	**Organism**	**PBS residues**	**Group**
			**(Figure**2**)**
4455142	*Lentinus sajor-caju*	EVDDHISFTARRGC	D
173523	*Schizophyllum commune*	EVDDHFSFTARRGL	D
74699315	*Ustilago maydis*	EVDDHLSFTARRGL	D
11229034	*Melampsora lini*	EVDDHISFTARRGL	D
55982602	*Cochliobolus heterostrophus*	EVDDHSSFTSRRGL	C
173523	*Schizosaccharomyces pombe*	STDDHAAFAAIKDL	B
3435	*Saccharomyces cerevisiae*	ETGDNSSYTAIQGL	B
170938	*Candida albicans*	ETGENSSYTSMKDL	B
77023508	*Verticillium tricorpus*	QNGDHASFTSRRGL	A
299296	*Phaeosphaeria nodorum*	QTGDHASFTSRRGL	A
58119500	*Phaeosphaeria avenaria*	QTGDHASFTSRRGL	A
170600	*Venturia inaequalis*	QTGDHASFTSRRGL	A
61678005	*Cercospora beticola*	QTGDHASFTSRRGL	A
30961893	*Monilinia fructicola*	QTGDHASFTSRRGL	A
166496	*Aspergillus flavus*	QTGDHASFTSRRGL	A
168105	*Emericella nidulans*	QTGDHASFTSRRGL	A
639766	*Ajellomyces capsulatus*	QTGDHASFTSRRGL	A
2852439	*Mycosphaerella pini*	QTGDHASFTSRRGL	A
1002511	*Botryotinia fuckeliana*	QTGDHASFTSRRGL	A
602578	*Erysiphe pisi*	QTGDHASFTSRRGL	A
1060942	*Penicillium digitatum*	QTGDHASFTSRRGL	A
6652864	*Pestalotiopsis microspora*	QTGDHASFTSRRGL	A
1263904	*Rhynchosporium secalis*	QTGDHASFTSRRGL	A
2718	*Epichloe typhina*	QTGDYASFTSRRGL	A
849160	*Gibberella fujikuroi*	QTGDYASFTSRRGL	A
2293	*Neotyphodium coenophialum*	QTGDYASFTSRRDL	A
32130590	*Gibberella zeae*	QTGDYASFTSRRGL	A
167300	*Glomerella graminicola*	QTGHHRSFTS-KGL	A
169400	*Pneumocystis carinii*	STGDHASFTSKRGL	A

Sequence numbers: 22-23, 26, 226, 229, 233, 236, 272, 276-278, 369-371.

**Table 3:** Plant β-tubulin sequences used in this work

**GI/NCBI Ref.**	**Organism**	**PBS residues**	**Group**
			**(Figure**2**)**
4455142	*Lentinus sajor-caju*	EVDDHISFTARRGC	C
1488052	*Daucus carota*	EVDDHGSFTSRRGL	C
460991	*Oryza sativa*	EVDDHASFTSRRGL	C
4415996	*Eleusine indica*	EVDDHASFTSRRGL	C
224106013	*Populus trichocarpa*	EVDDHASFTSRRGL	C
1403143	*Cicer arietinum*	EVDDHASFTSRKNL	C
244539475	*Lotus japonicus*	EVDDHASFTSRKGL	C
153799899	*Eucalyptus grandis*	EVDDHASFTSRKGL	C
255564502	*Ricinus communis*	EVDDHASFTSRKGL	C
312989	*Glycine max*	EVDDHASFTSRKGL	C
223018283	*Citrus maxima*	EVDDHASFTSRKGL	C
295851	*Zea mays*	EVADHASFTSRHGL	C
609270	*Solanu tuberosum*	EVDDHASFTSRTGL	C
77963735	*Solanum lycopersicum*	EVDDHASFTSRTGL	C
40036995	*Nicotiana attenuata*	EVDDHASFTSRTGL	C
1743277	*Hordeum vulgare*	EVDDHASFTSRTGL	C
14331109	*Medicago sativa*	EVDDHTSFTSRTGL	C
145388977	*Capsicum annuum*	EVDDHASFTSRTGL	C
37038246	*Physcomitrella patens*	EVEDHASFTSRTGL	C
402636	*Lupinus albus*	EVADHASFTSRTGL	C
20758	*Pisum sativum*	EVADHASFTSRTGL	C
20148289	*Arabidopsis thaliana*	EVADHASFTSRTGL	C
51988178	*Setaria viridis*	EVADHASFTSRTGL	C
205326619	*Prunus salicina*	EVADHASFTSRTGL	C
5668669	*Zinnia elegans*	EVTDHASFTSRTGL	C
296498	*Anemia phyllitidis*	EVTDHASFTSRVGL	C
4098333	*Triticum aestivum*	EVGDHASFTSRVGL	C
19569609	*Gossypium hirsutum*	EVADHASFTSRIGL	C

Sequence numbers: 22-23, 26, 226, 229, 233, 236, 272, 276-278, 369-371.

**Table 4:** Protist β-tubulin sequences used in this work

**GI/NCBI Ref.**	**Organism**	**PBS residues**	**Group**
			**(Figure**2**)**
155874	*Babesia bovis*	EVDDHASFTSRKGL	C
29420520	*Babesia microti*	EVDDHASFTSRKGL	C
295762	*Plasmodium falciparum*	EVDDHASFTSRKGL	C
4079637	*Tetrahymena pyriformis*	EVDDHASFTSRKGL	C
161737	*Tetrahymena thermophila*	EVDDHASFTSRKGL	C
6007456	*Stylonychia mytilus*	EVDDHASFTSRKGL	C
9309	*Euplotes octocarinatus*	EVDDHASFTSRKGL	C
2155306	*Chlamydomonas incerta*	EVDDHASFTSRKGL	C
167456	*Chlamydomonas reinhardtii*	EVDDHASFTSRKGL	C
166302	*Achlya klebsiana*	EVDDHASFTSRKGL	C
68128910	*Leishmania major*	EVDDHASFTSRKGL	C
6652866	*Pythium ultimum*	EVDDHASFTSRKGL	C
639490	*Eimeria tenella*	EVDDHASFTSRKGL	C
161939	*Toxoplasma gondii*	EVDDHASFTSRKGL	C
23481527	*Plasmodium yoelii yoelii*	EVDDHASFTSRKGL	C
290685	*Moneuplotes crassus*	EVDDHASFTSRKGL	C
38520885	*Paramecium tetraurelia*	EVDDHASFTSRKGL	C
295443942	*Palpitomonas bilix*	EVDDHASFTSRKGL	C
238617571	*Leucocryptos marina*	EVDDHASFTSRKGL	C
302849658	*Volvox carteri*	EVDDHASFTSRKGL	C
135500	*Trypanosoma brucei rhodesiens*	EVDDHASFTSRKGL	C
206598211	*Bodo saltans*	EVDDHASFTSRKGL	C
159416	*Leishmania mexicana*	EVDDHASFTSRKGL	C
135494	*Polytomella agilis*	EVDDHASFTSRKGL	C
829213	*Naegleria gruberi*	EVDDHISFTSRRGL	C
8926601	*Thalassiosira weissflogii*	EVDDHACYTSRKGL	C
2951981	*Phytophthora cinnamomi*	EVDDHASFTSRQGL	C
29150706	*Porphyra yezoensis*	EVEDKGSYSADTGE	D
29539330	*Cyanidioschyzon merolae*	EVEDKGSLTATKGL	D
1067176	*Porphyra purpurea*	EVEDKGSYSADTGE	D

Sequence numbers: 22-23, 26, 226, 229, 233, 236, 272, 276-278, 369-371.

### Paclitaxel indifference of yeast and βIII-tubulin isotype reflected on the first two principal components

**Figure 3:**
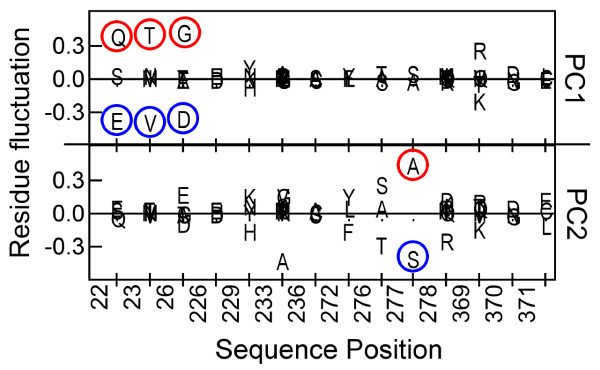
Variations of amino acid composition (from the average composition of the entire data set) at the PBS, as principal component 1 (PC1) and principal component 2 (PC2) vectors deviate from their respective mean (zero) values.

The identities of PBS residues were explored along PC1 or PC2 axes. Amino acid variations along PC1 and PC2 axes are shown in Figure 3. Among the five residue mutations that altered yeast tubulin affinity towards paclitaxel (A19K, T23V, G26D, N229H, and Y272F), two (T23V and G26D) are captured by the PC1 axis. Two other residue variations (N229H, and Y272F) get reflected along the PC2 axis (the fifth residue, A19K, is not part of the PBS). The only residue that makes the βIII-isotype unique is Ala277 (in place of Ser). This variation is reflected along the PC2 axis. It is noteworthy that the PCA was performed without the inclusion of the β-tubulin isotypes. Yet, sequence variations at the PBS in the isotype sequences (position 277) are reflected in one of the two PC axes. This shows that the two PC axes are quite robust. The Ala/Ser variation at position 277 is naturally present, not only in isotypes βIII and βVI, but also in β-tubulin across eukaryotes. In the next sections we provide structural arguments as to why members of groups A, B and D might be paclitaxel-insensitive.

### Structural consequences of Val23Thr and Asp26Gly mutation

**Figure 4:**
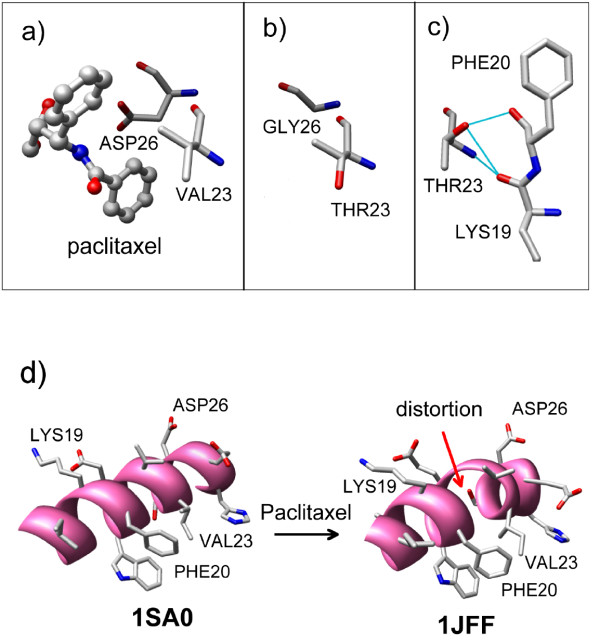
**Paclitaxel-bound (pdb ID:1JFF) and paclitaxel-free (pdb ID: 1SA0) β-tubulin. a)** Disposition of Val23, Asp26 and paclitaxel in paclitaxel-bound β-tubulin (pdb ID:1JFF). **b)** Same as panel **(a)** with two *in silico* mutations: Val23Thr and Asp26Gly. The rotameric state of Val23 in the original pdb file (1JFF) was maintained during the mutation. **c)** Identical to panel **(b)** except that the side-chain of Thr23 has been changed to a new rotameric state. In the new rotameric state, the sidechain of Thr23 can form a H-bond with the backbone carbonyl oxygen atoms of Phe20 and Lys19. **d)** An α-helix in β-tubulin (containing residues 19, 20 and 23) as found in two crystal structures: 1SA0 (paclitaxel-free) and 1JFF (paclitaxel-bound).

Compared to group C that contains paclitaxel-sensitive β-tubulin sequences, Val23Thr and Asp26Gly mutations characterize groups B and A (positive PC1 values). If these mutations are indeed responsible for paclitaxel-insensitivity, what is the structural mechanism behind paclitaxel-resistance? In Figure 4a paclitaxel is shown with Asp26 and Val23 as found in the structure 1JFF. The structural consequences of Asp26Gly and Val23Thr mutations are shown in Figure 4b. The large side chain of Asp molecule strongly interacts with 3′-phenyl group (Figure 4a) of paclitaxel. Asp26Gly mutation will clearly have a major effect since the Asp side-chain makes several contacts with paclitaxel that will be lost. However, the effect of Val23Thr mutation on paclitaxel-sensitivity of β-tubulin is not apparent from Figure 4a. To fully understand the effect of Val23Thr mutation, one needs to examine alternate rotameric states of Thr23. In the new rotameric state (Figure 4c) the side-chain of Thr23 can form a H-bond with the backbone carbonyl atoms of Lys19 and Phe20. Although this new interaction is a little distant from paclitaxel, it can have subtle but important effect on paclitaxel-binding. As shown in Figure 4d, Lys19, Phe20, Val23, and Asp26 β-tubulin is part of a helix in paclitaxel-bound (pdb code: 1JFF) as well as in paclitaxel-free (pdb code: 1SA0) structure. However, paclitaxel-binding distorts the helix. Upon Val23Thr mutation, the side-chain hydroxy group of Thr can potentially form a H-bond with the backbone oxygen atom of Phe 20 and Lys 19 (Figure 4c). Therefore, the observed helix distortion is harder to achieve when Val gets mutated to Thr at position 23. This extra H-bond formed by Thr 23 will be detrimental to paclitaxel-binding. In other words, Val23Thr mutation can affect paclitaxel sensitivity in an indirect manner.

### Structural consequences of Ser277Ala mutation

**Figure 5:**
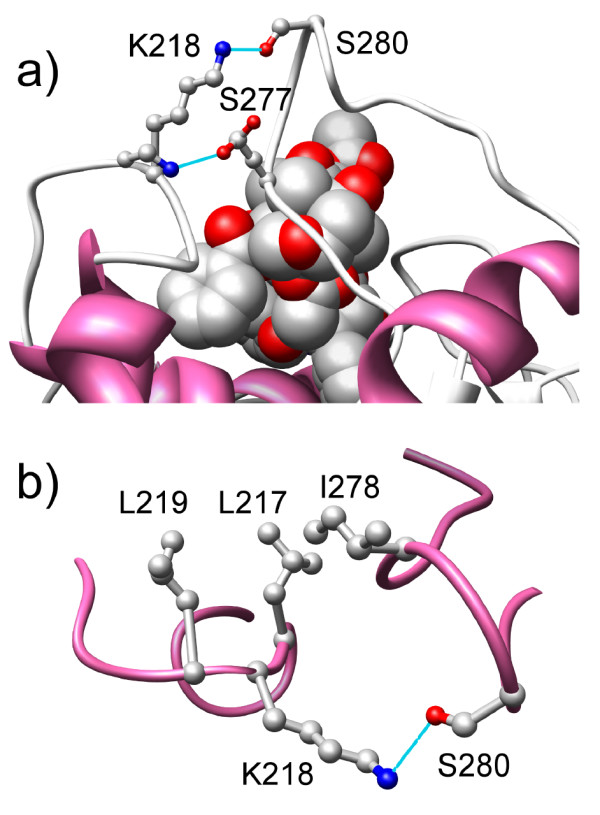
**Loop-loop interactions in paclitaxel-bound animal β-tubulin (pdb ID: 1JFF). a)** The side-chain of Ser277 is shown in two rotameric states — the first rotamer (as found in 1JFF) lacks Ser277-Lys218 interaction while the second rotamer (generated *in silico*) shows Ser277-Lys218 H-bond (blue line) interaction. The interaction between Ser280 and Lys218 is present in the crystal structure. **b)** Disposition of loops of panel **(a)** with two *in silico* mutations (S277A and K278I; 277A and 278I are present in yeast β-tubulin) exhibiting a three-center hydrophobic interaction (L219-L217-I278).

Serine is present at position 277 in βIII and βVI isotypes (the βVI isotype also has amino acid changes at other positions) while all other isotypes contain Ala at this position (Figure 1). Here we explore mechanisms by which Ser277Ala mutation can give rise to paclitaxel resistance. This amino acid change was reflected in the PC2 axis. As shown in Figure 5, Ser277 is present in a flexible loop around the bound paclitaxel in 1JFF and is proximal to another loop containing Lys218. That the loops are flexible and become more ordered upon binding paclitaxel is clear from the fact that in the paclitaxel-free state (pdb ID:1SA0), residues 278 to 285 in one of the loops is disordered in β-tubulin. Upon paclitaxel binding (pdb ID: 1JFF) this loop becomes ordered. Interactions between the two loops present in 1JFF are shown in Figure 5a. In the paclitaxel-bound state, the side chain hydroxyl group of Ser280 forms a H-bond with the side chain nitrogen of Lys218 (note that residue 280 is disordered in 1SA0 and so no such interaction is present). This indicates that interaction of the two loops is important for paclitaxel binding. Ser277 does not participate in loop-loop interaction in the paclitaxel-bound structure. Side-chain rotamers are often missed in protein structures, especially in structures like 1JFF, which was determined by electron diffraction and has a rather low resolution (3.5 Å). So we altered (*in silico*) the rotameric state of Ser277 to examine if that would introduce any loop-loop interaction. Indeed, as shown in Figure 5a, one rotameric state of Ser277 showed side-chain H-bonding to the backbone amide of Lys 218. This indicates that upon Ser277Ala mutation, the possibility of formation of an additional H-bond formation would be lost. This loss of additional H-bond energy may be indirectly responsible for resistance of isotype βIII by directly affecting loop-loop interaction.

### Why mutated yeast β-tubulin is paclitaxel-sensitive even with Ala at position 277

*S. cerevisiae* β-tubulin, a member of group B in Figure 2a, is paclitaxel-insensitive. According to the major amino acid changes among the quadrants, this is due to the presence of: i) Ala instead of Ser at position 277, and, ii) Thr and Gly (instead of Val and Asp) at positions 23 and 26 respectively. Paclitaxel-sensitivity in *S. cerevisiae* was achieved with three single mutations: Thr23Val, Gly26Asp and Tyr272Phe. However, Ala277 was unaltered in that experiment. According to the arguments presented above, β-tubulin of the triple mutant would still be paclitaxel insensitive since Ala 277 (instead of Ser 277) will destabilize the loop-loop interaction. The reason why this is not the case becomes clear if one looks at the sequence of yeast β-tubulin (Table 2). The presence of Ile in place of Arg/Lys (conserved in all other members except the three quadrant B sequences) is conspicuous at position 278, a position that follows Ala277. If one examines the tubulin structure, Ile278 is in proximity to Leu217 and Leu 219 (Figure 5b) allowing the formation of a three-center hydrophobic staple, stabilizing the loop-loop interaction. In other words, although yeast β-tubulin lacks the potential loop-loop interaction mediated by Ser277-Lys218 H-bond, its absence is compensated by an alternate potential loop-loop interaction mediated by a three-centered hydrophobic staple Ile-Leu-Leu (278-217- 219).

### Consequences of mutations in yeast β-tubulin at positions 19, 229 and 272

**Figure 6:**
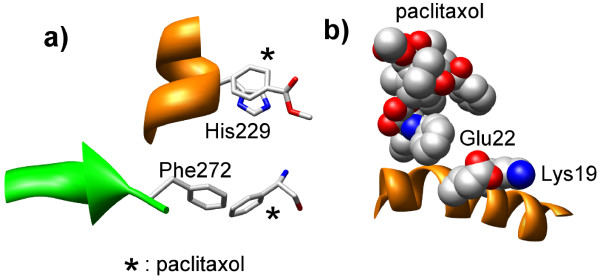
**Disposition of a) paclitaxel, Phe272 and His229, and b) paclitaxel, Lys19 and Glu22 in paclitaxel-bound β-tubulin (pdb ID: 1JFF).** Although Lys19 is far from paclitaxel, its role in intra-helix interaction is emphasized by its proximity to Glu22.

Of the five mutations that transformed yeast β-tubulin from paclitaxel non-binder to paclitaxel-binder, we have discussed structural consequences of only two: T23V and G26D. Here we look at the other three mutations. Interactions of residues His229 and Phe272 with paclitaxel in 1JFF are shown in Figure 6a. While His participates in stacking interactions, Phe participates in an edge-edge interaction with paclitaxel. The His229Asn mutation will clearly disrupt the stacking interaction and affect paclitaxel-binding affinity. However, it seems that Phe272Tyr mutation may not play an important role in disrupting paclitaxel-β-tubulin interaction. However, only experiments with single mutants can clearly address the importance of the F272Y mutation. We did not consider residue 19 in our work since it was not part of the PBS. In fact, as shown in Figure 6b, residue 19 is quite distant from paclitaxol in 1JFF. Nonetheless, its importance can be gauged if one considers ion-pair interaction between Lys19 and Glu22, stabilizing a helix proximal to paclitaxel. To summarize, K19A mutation can directly affect the stabilization of a helix proximal to paclitaxol and affect binding. The H229N mutation can disrupt an important stacking interaction and affect binding. The F272Y mutations may not play an important role in modulating paclitaxel binding.

## Discussion

Paclitaxel is an important chemotherapeutic drug that binds β-tubulin and modulates microtubule formation and dynamics. The paclitaxel-binding site on β-tubulin has been identified from the structure of paclitaxel-bound β-tubulin. In addition, a large number of β-tubulin sequences across all four eukaryotic families are known. Using the paclitaxel-bound β-tubulin structure and the β-tubulin sequence database we have attempted to rationalize two apparently unrelated experimental data, relevant to paclitaxel-β-tubulin binding: 1) induction of paclitaxel resistance upon over expression of human tubulin βIII-isotype [11-17], 2) yeast β-tubulin, which otherwise does not bind paclitaxel, becomes paclitaxel-sensitive upon mutation at five positions [20,21].

Using the paclitaxel-bound β-tubulin structure and multiple sequence alignment, paclitaxel-binding site residues in β-tubulin across eukaryotic families were identified. Subsequently PCA was performed in the sequence sub-space comprising the binding site. By projecting the binding site residues onto the first two PC vectors we checked if the sequences from fungal tubulin formed a cluster, since available experimental data suggests that only members of the fungi family are paclitaxel-insensitive. However, no clear fungi family-specific clustering was obtained. In a previous study a similar approach yielded a very clear family-specific clustering of tubulin sequences, correlating with experimental data, where the focus was on the binding site of another anti-mitotic drug, colchicine [31].

Of the three mutations in yeast β-tubulin that rendered it paclitaxel-sensitive, two (T23V and G26D) were captured by the PC1 axis and one (Y270F) was captured by the PC2 axis. The distribution of the three mutations in two axes allowed us to define four clusters in the PC1-PC2 plane. Most animals and protists, including all plants, were found in one cluster (C). Representative members of these families are known to be paclitaxel-sensitive. However quadrant C also contained one member of the fungi family, *C. heterostrophus*. Close inspection of the sequences of members of cluster C (Table 1) shows that *C. heterostrophus* is unique among members of the C cluster. In *C. heterostrophus* the sequence position 233 is occupied by Ser, while for all other members, this position is occupied by Ala/Leu/Met/Val/Gly/Ile. Therefore the sole member of the fungi family in this cluster is unique and might actually be paclitaxel-insensitive. The rest of the members of the fungi family were distributed in the other three clusters, uniquely characterized by the presence of mutations responsible for paclitaxel-insensitivity. Structural consequences of mutations leading to paclitaxel-insensitivity in yeast tubulin and members present in clusters A, B and D of the PC1-PC2 projection were examined and it was shown that while some mutations will have a direct consequence, for others the consequences of mutations on paclitaxel-binding may be indirect.

The only position in the PBS where a residue change correlated with paclitaxel-insensitivity change in human β-tubulin isotypes (Ser277Ala mutation) was captured by PC2 axis even though tubulin isotype sequences were excluded in the PCA. This is a striking result and shows that the two PC axes not only capture inter-species sequence variations they also naturally capture intra-species sequence changes at critical amino acid positions in the PBS. The structural role of Ser277 has recently been studied by docking and molecular dynamics studies of βI and βIII tubulin isotypes and their complexes with antimitotic agents, where it was shown that Ser277 plays a crucial role in intra-loop interaction critical for paclitaxel binding [32]. We showed that Ser277 also plays an important role in inter-loop interaction that can affect paclitaxel binding. Despite being a critical residue, Ser277Ala mutation can still allow paclitaxel binding, provided there are alternate mechanisms of loop-loop interaction, as evident from mutated yeast tubulin where a Ser277Ala mutation is compensated by Arg278Ile mutation, giving rise to a three-center hydrophobic staple. In addition, human βVI tubulin isotype, which also contains Ala277, may actually be paclitaxel sensitive due to other compensatory mutations (Arg278Gln, Ala233Leu).

## Conclusions

In summary, we have rationalized two apparently unrelated experimental data — differential paclitaxel binding among mammalian β-tubulin isotypes and that between wild type and yeast β-tubulin mutated at six positions — in terms of two collective sequence vectors, PC1 and PC2, spanning the PBS. PCA has previously been successful in predicting protein functional residues [33] and rationalizing the differential colchicine-binding of animal-tubulins [31]. The present analysis extends the applicability of PCA to paclitaxel-tubulin interaction with valuable insights and specific predictions.

## Methods

A total of 125 β-tubulin (animal: 38, fungi: 29; protists: 30; plant: 28) sequences were used in this work. The sequence accession numbers and the names of the corresponding organisms are given in Tables 1,2,3,4. The structures for paclitaxel-bound (PDB ID: 1JFF) [23] and paclitaxel-free (PDB ID: 1SA0) [34] tubulin were downloaded from the protein data bank [35]. Structural analysis was performed using the program Chimera [36]. The primary paclitaxel binding site (PBS) was defined by residues in 1JFF that were within 5 Å of paclitaxel (distance between atoms of paclitaxel and any heavy atom of tubulin). Amino acid residues within 5 Å from the bound paclitaxel molecule are given in Figure 1. Multiple sequence alignments were performed using the program ClustalW [24] to identify PBS residues in all β-tubulin sequences corresponding to that defined in 1JFF.

Subsequently, Principal Component Analysis (PCA) was performed in the multi-dimensional sequence-space of PBS, as previously described [31]. Each sequence position was represented by a binary-vector (of length 21), where the first twenty elements represent the occurrence of a particular amino acid (1 for presence and 0 for absence), and the last element represents the presence of a gap. For example, Ala is represented by {1, 0, 0, 0, 0, 0, 0, 0, 0, 0, 0, 0, 0, 0, 0, 0, 0, 0, 0, 0, 0} and a gap is represented by {0, 0, 0, 0, 0, 0, 0, 0, 0, 0, 0, 0, 0, 0, 0, 0, 0, 0, 0, 0, 1}. The PBS residues (22 residue positions, of which 14 positions showed amino acid variations) from each β-tubulin sequence (a total of M sequences) was represented by a binary-vector {*a*_*ik*_} where the *i*-th index represents a particular β-tubulin sequence (*i* = 1, M) and the *k*-th index runs from 1 to N (N = 14 × 21). The N × N variance-covariance matrix was prepared as:

(1) $$C_{\mathrm{kl}}=\sum_{i=1}^{M}\left(a_{\mathrm{ik}}-{\bar{a}}_{k}\right)\left(a_{\mathrm{il}}-{\bar{a}}_{l}\right)/(M-1)$$

where *ā*_*k*_ and *ā*_*l*_ are given by:

(2) $$\begin{array}{l} {\bar{a}}_{k}=\sum_{i=1}^{M}a_{\mathrm{ik}}/M\\ {\bar{a}}_{l}=\sum_{i=1}^{M}a_{\mathrm{il}}/M \end{array}$$

In Eq. 1, the denominator (M - 1), instead of M, is used to yield an unbiased estimator of the (co)variance. The variance-covariance matrix was diagonalized yielding N principal component vectors (eigen vectors ${\vec{V}}_{k}$; *k* = 1, N) and N eigen values (mean square variation associated with each eigen vector) *λ*_*k*_ (*k* = 1, N). The projection of the *i*-th β-tubulin PBS sequence on the *k*-th PC vector ${\vec{V}}_{k}$ was calculated from the dot product of *i*-th sequence vector {$a_{i1},a_{i2}\ldots a_{\mathrm{iN}}$} and the *k*-th PC vector {$v_{k1},v_{k2}\ldots v_{\mathrm{kN}}$}.

## Competing interests

The authors declare no competing interests.

## Authors’ contributions

GB and BB conceived of the study, GB and LD participated in its design and performed the sequence analysis, and all authors analyzed the data and GB drafted the manuscript. All authors read and approved the final manuscript.
